# The Transcriptomic Analysis of NSC-34 Motor Neuron-Like Cells Reveals That Cannabigerol Influences Synaptic Pathways: A Comparative Study with Cannabidiol

**DOI:** 10.3390/life10100227

**Published:** 2020-10-01

**Authors:** Agnese Gugliandolo, Serena Silvestro, Luigi Chiricosta, Federica Pollastro, Placido Bramanti, Emanuela Mazzon

**Affiliations:** 1 IRCCS Centro Neurolesi “Bonino-Pulejo”, Via Provinciale Palermo, Contrada Casazza, 98124 Messina, Italy; agnese.gugliandolo@irccsme.it (A.G.); serena.silvestro@irccsme.it (S.S.); luigi.chiricosta@irccsme.it (L.C.); placido.bramanti@irccsme.it (P.B.); 2 Department of Pharmaceutical Sciences, University of Eastern Piedmont, Largo Donegani 2, 28100 Novara, Italy; federica.pollastro@uniupo.it

**Keywords:** Cannabidiol, Cannabigerol, Glutamatergic synapses, GABAergic synapses, motor neuron-like cells, transcriptomic analysis

## Abstract

More than 120 cannabinoids were isolated from *Cannabis sativa*. In particular, Cannabidiol (CBD) and Cannabigerol (CBG) represent the two most studied non-psychoactive cannabinoids. However, CBG is less studied and less data are available on its biological properties and influence on synaptic transmission. On the contrary, CBD is already known to modulate brain excitatory glutamate, inhibitory γ-aminobutyric acid (GABA) and dopamine neurotransmission. In this study, using Next-Generation Sequencing (NGS) technology, we evaluated how CBG (1 or 5 µM) and CBD (1 or 5 µM) influence the transcriptome of the main neurotransmission pathways in NSC-34 motor neuron-like cells. At first, we evaluated that CBG and CBD were not cytotoxic and decreased the expression of pro-apoptotic genes. CBG and CBD are able to influence the expression of the genes involved in glutamate, GABA and dopamine signaling. Interestingly, the transcriptional changes induced by CBG were similar compared to CBD.

## 1. Introduction

**Figure 1:**
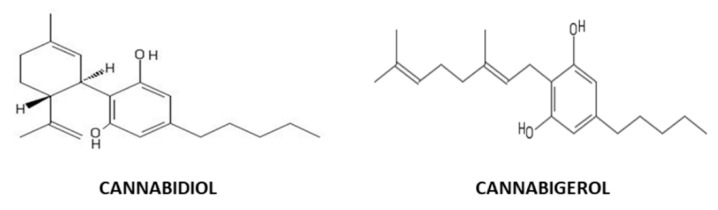
Cannabidiol (CBD) and cannabigerol (CBG) chemical structures, two non-psychotropic phytocannabinoids isolated from *Cannabis sativa*.

*Cannabis sativa* is a plant belonging to the *Cannabaceae* family and represents a reservoir of cannabinoids, knowns for their biological properties [1]. Delta-9-tetrahydrocannabinol (Δ^9^-THC) is the most abundant psychotropic compound. However, other phytocannabinoids such as Cannabidiol (CBD) and Cannabigerol (CBG) can be isolated from *Cannabis sativa* (Figure 1). CBG and CBD have the advantage of being two non-psychotropic phytocannabinoids that show different health-promoting effects.

CBD and CBG exert their activity through interaction with multiple molecular sites. These phytocannabinoids interact with cannabinoid receptors (CBs) coupled to G proteins, located in regions of the brain associated with important neurological processes [2]. However, these two cannabinoids are characterized by different action profiles. In particular, CBD shows a low affinity for CBs, acting as a negative allosteric modulator of CB1 receptors and as an inverse agonist of CB2 receptors [3]. Instead, CBG is a partial CB1 and CB2 receptor agonist. Moreover, CBG and CBD are also capable of regulating G protein-coupled receptors (GPCRs) [4]. In addition, CBD is a partial agonist at dopamine D2 receptors [5].

Although these two phytocannabinoids own multiple biological targets, an important aspect could be their influence on synaptic pathways. Glutamate, GABA and dopamine are among the main neurotransmitters in the brain. In particular, glutamate and GABA are involved in excitatory and inhibitory neurotransmission, respectively [6,7]. Glutamate, an excitatory neurotransmitter, plays a key role in synaptic transmission, synaptic plasticity, neuronal migration, neuronal excitability, long-term potentiation and long-term depression [8]. GABA, an inhibitory neurotransmitter, is involved in the modulation of synaptic transmission, in the promotion of neuronal development, in the prevention of insomnia and depression, in motor cortical plasticity and in learning [9,10,11,12]. Dopamine is a monoamine catecholamine neurotransmitter that regulates motor functions, motivation, cognition, emotion and neuroendocrine secretion. Regarding these synaptic pathways, less studies are available for CBG, while only for CBD there are some data. Indeed, it is known that CBD promotes the transmission of glutamate and GABA [13,14]. CBD can also functionally regulate dopaminergic transmission [15,16]. Then, there is a great interest to study and deeply analyze the biological properties of CBG.

Using the NGS analysis, we aim to evaluate how CBG and CBD (1 or 5 µM) influence the expression profile of the genes involved in neurotransmission pathways in NSC-34 motor neuron-like cells.

## 2. Results

### 2.1. Cell Viability

**Figure 2:**
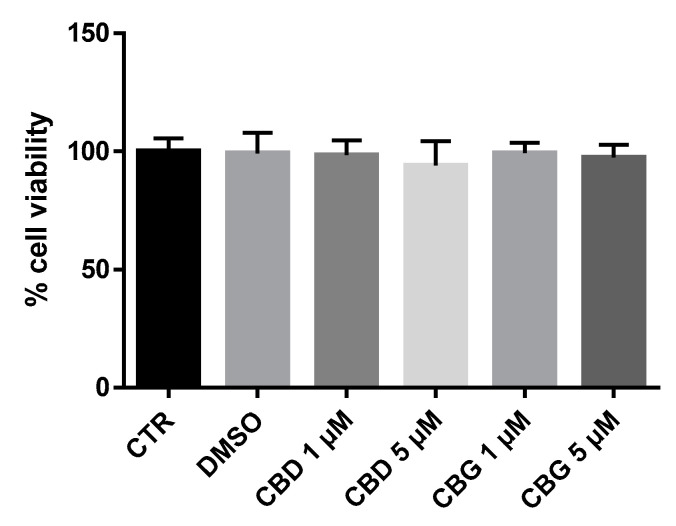
Cell viability in NSC-34 motor neuron-like cells exposed for 24 h to different cannabigerol (CBG) and cannabidiol (CBD) concentrations (1 or 5 µM). In all doses, no significant variation on cell viability was observed compared to the control group (*p* > 0.05). Data were expressed as the mean (± SD).

In order to assess the cytotoxic effects of CBD and CBG, NSC-34 motor neuron-like cells were exposed to a 1 or 5 µM concentration of CBD or CBG, and the cell viability was evaluated. CBD or CBG at all doses tested (Figure 2) did not show cytotoxicity, as demonstrated by Thiazolyl Blue Tetrazolium Bromide (MTT) assay. Indeed, the percentage of viable cells was relatively similar to the control cells (CTR-NSC-34) in all groups. We also analyzed NSC-34 cells incubated with dimethyl sulfoxide (DMSO < 0.1%), and no cytotoxicity was observed.

### 2.2. Differential Expressed Genes Inspection

**Figure 3:**
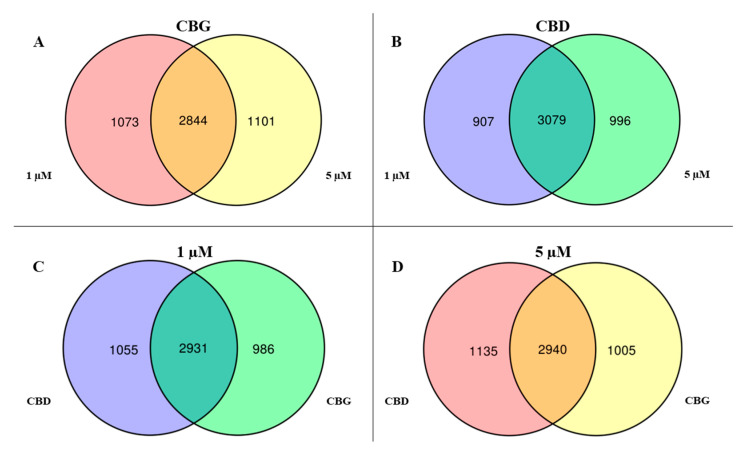
Venn Diagram. For both the (**A**) CBG and (**B**) CBD compound, the central area highlights the amount of genes that are statistically differentially expressed between CTR-NSC-34 and dose 1 and 5 µM of CBG (2844) or CBD (3079). The right areas of both the diagrams highlight the genes statistically differentially expressed between CTR-NSC-34 and dose 5 µM of CBG (1101) or CBD (996) that are not significant at dose 1 µM. Conversely, the left area of the diagrams shows the genes that are statistically differentially expressed between CTR-NSC-34 and dose 1 µM of CBG (1073) or CBD (907) that are not significant at dose 5 µM. For both the (**C**) 1 and (**D**) 5 µM doses, the central area highlights the amount of genes that are statistically differentially expressed between CTR-NSC-34 and dose 1 µM of CBD or CBG (2931) or 5 µM of CBG or CBD (2940). The left areas of both the diagrams highlight the genes statistically differentially expressed exclusively between CTR-NSC-34 and dose 1 µM of CBD (1055) or 5 µM CBD (1135). Conversely, the right area of the diagrams shows the genes that are statistically differentially expressed exclusively between CTR-NSC-34 and dose 1 µM of CBG (986) or 5 µM CBG (1005).

We carried out the RNA-seq analysis for each of the two different doses (1 and 5 µM) of the CBG and CBD compared to the control. The NGS analysis revealed in the final demultiplexed FastQ file 6,008,356 for CTR-NSC-34, 8,886,408 reads for CBG at 1 µM and 10,341,470 for CBG at 5 µM; 10,245,166 reads for CBD at 1 µM and 10,286,312 reads for CBD at 5 µM. To describe the transcriptomic analysis, we depicted a Volcano Plot for each of the doses of the CBG (Supplementary Figure S1) and of the CBD treatment (Supplementary Figure S2). We then applied the cut-off on the q-value (0.05) and on the fold change (0.7). Specifically, we identified 3917 genes differentially expressed at 1 µM of CBG and 3945 at 5 µM. As showed in the Venn diagram in Figure 3A, both the doses of CBG modulated 2844 genes. Instead, 1101 genes were modulated exclusively at dose 5 µM, while 1073 genes at dose 1 µM. About CBD, 3986 genes were differentially expressed at 1 µM and 4075 at 5 µM. Again, as shown in Figure 3B, we observed 3079 genes modulated simultaneously by doses of 5 and 1 µM. The amount of 5 µM of CBD also modulated 996 genes exclusively, while CBD 1 µM 907 genes. We also compared CBG and CBD for each concentration (Figure 3C,D). Regarding the 1 µM dose, CBG exclusively modulated 986 genes, while CBD, 1055. However, the majority of the genes (2931) were commonly modulated between CBD and CBG 1 µM. Regarding the 5 µM dose, CBG modulated 1005 genes, while CBD, 1135. Instead, 2940 genes were modulated by both CBG and CBD at the same dose of 5 µM.

**Figure 4:**
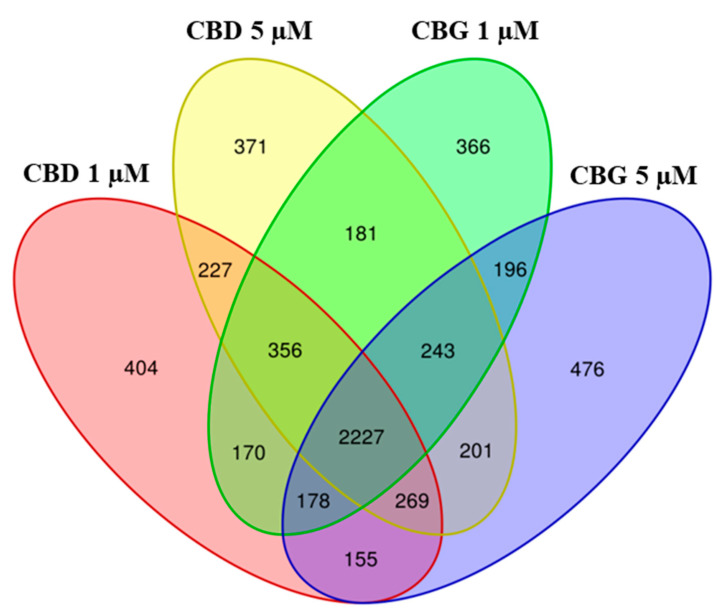
Venn Diagram. The genes differentially expressed in CBD 1 µM, CBD 5 µM, CBG 1 µM and CBG 5 µM were evaluated. The comparison of all the samples shows how many genes are differentially expressed between the different compounds at each concentration. Each intersection of the different samples shows the number of genes in common, exclusively between those groups and not with the others.

We also compared CBG and CBD at both the concentrations (Figure 4). Interestingly, the highest number of genes was commonly modulated by both compounds at both concentrations (2227 genes).

To confirm the non-cytotoxicity of CBG or CBD, we analyzed the genes involved in the apoptosis (Table 1). We observed that the anti-apoptotic genes thymoma viral proto-oncogene 1 (*AKT1*), activating transcription factor 4 (*ATF4*) and BCL2-like 1 (*BCL2L1*) were upregulated, while the pro-apoptotic genes eukaryotic translation initiation factor 2 alpha kinase 3 (*EIF2AK3*), endoplasmatic reticulum (ER) to nucleus signaling 1 (*ERN1*), mitogen-activated protein kinase kinase kinase 5 (*MAP3K5*)*,* poly(ADP-ribose) polymerase family, member 4 (*PARP4*)*,* caspase 6 (*CASP6*) and caspase 8 (*CASP8*) were downregulated. Interestingly, *CASP6* was significantly expressed only in CBD at 5 µM, while *CASP8* only in CBG at 1 µM and in CBD at 5 µM.

### 2.3. KEGG Pathway Analysis

**Figure 5:**
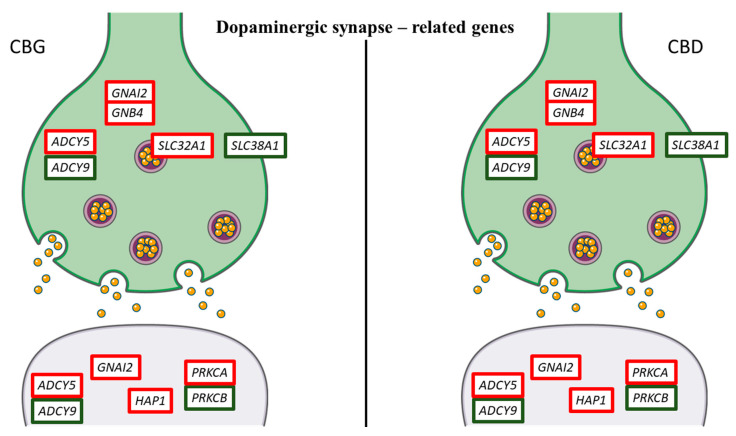
Genes related to dopaminergic synapse differentially regulated by CBG and CBD. Genes upregulated are represented with the red edges, while the genes downregulated have green edges. Interestingly, CBG and CBD showed the same pattern of gene expression. The figure was created using the vector image bank of Servier Medical Art by Servier [17] and licensed under a Creative Commons Attribution 3.0 Unported License [18].

**Figure 6:**
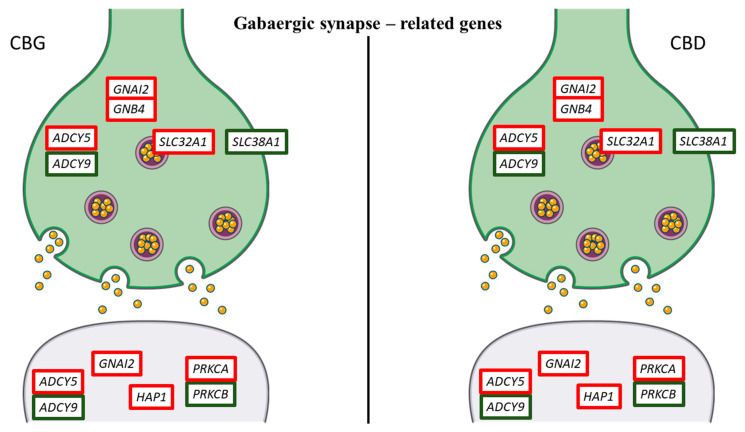
Genes related to GABAergic synapse differentially regulated by CBG and CBD. Genes upregulated are represented with the red edges, while the genes downregulated have green edges. Interestingly, CBG and CBD showed the same pattern of gene expression. The figure was created using the vector image bank of Servier Medical Art by Servier [17] and licensed under a Creative Commons Attribution 3.0 Unported License [18].

**Figure 7:**
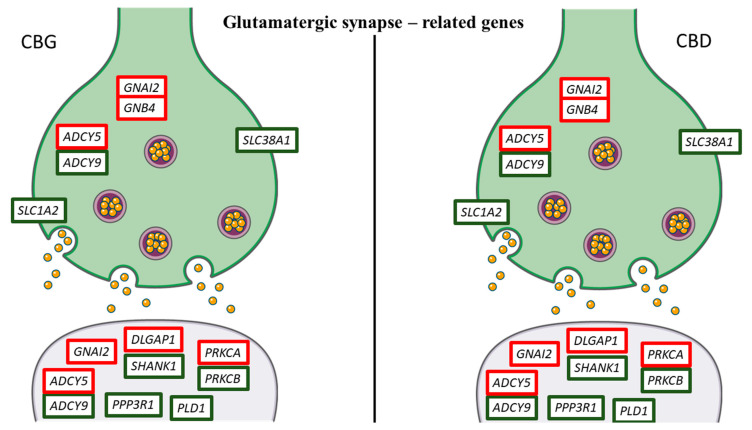
Genes related to glutamaergic synapse differentially regulated by CBG and CBD. Genes upregulated are represented with the red edges, while the genes downregulated have green edges. Interestingly, CBG and CBD showed the same pattern of gene expression. The figure was created using the vector image bank of Servier Medical Art by Servier [17] and licensed under a Creative Commons Attribution 3.0 Unported License [18].

In addition, in order to highlight the effects of CBG and CBD in NSC-34 moto neuron-like cells, we inspected the genes that take part in the nervous system category in KEGG. We studied the most representative genes related to “Dopaminergic synapse”, “GABAergic synapse” and “Glutamatergic synapse” pathways (mmu04728, mmu04727 and mmu04724, respectively). In Table 2, we put the 23 genes included in the pathways. Ten of these genes, that are adenylate cyclase 5 (*ADCY5*), *AKT1*, *ATF4*, DLG associated protein 1 (*DLGAP1*), dopamine receptor D4 (*DRD4*), guanine nucleotide binding protein (G protein), alpha inhibiting 2 (*GNAI2*), guanine nucleotide binding protein (G protein), beta 4 (*GNB4*), huntingtin-associated protein 1 (*HAP1*), protein kinase C, alpha (*PRKCA*), solute carrier family 32 (GABA vesicular transporter), member 1 (*SLC32A1*), were upregulated in all the doses both for CBG and CBD. Conversely, thirteen genes, that are adenylate cyclase 9 (*ADCY9*), calcium/calmodulin-dependent protein kinase II, beta (*CAMK2B*), circadian locomotor output cycles kaput (*CLOCK*), cAMP responsive element binding protein 1 (*CREB1*), dopamine receptor D2 (*DRD2*), guanine nucleotide binding protein, alpha stimulating, olfactory type (*GNAL*), phospholipase D1 (*PLD1*), protein phosphatase 3, regulatory subunit B, alpha isoform (calcineurin B, type I) (*PPP3R1*), protein kinase C, beta (*PRKCB*), SH3 and multiple ankyrin repeat domains 1 (*SHANK1*), solute carrier family 1 (glial high affinity glutamate transporter), member 2 (*SLC1A2*), solute carrier family 18 (vesicular monoamine), member 1 (*SLC18A1*), solute carrier family 38, member 1 (*SLC38A1*) were always downregulated. Moreover, *ADCY5*, *GNAI2*, *GNB4*, *PRKCA* and *PRKCB* were in common to all the studied pathways. *AKT1*, *ATF4*, *CAMK2B*, *CLOCK*, *CREB1*, *DRD2*, *DRD4*, *GNAL* and *SLC18A1* are genes that take part only in the “Dopamine synapse” (Figure 5), while *HAP1* and *SLC32A1* only in the “GABAergic synapse” (Figure 6). Instead, the genes *DLGAP1*, *PLD1*, *PPP3R1*, *SHANK1* and *SLC1A2* are exclusive “Glutamatergic synapse”-related genes (Figure 7).

## 3. Discussion

Non-psychotropic cannabinoids are receiving increasing attention due to their biological properties.

In this study, the NSC-34 motor neuron-like cells were treated with CBG (1 or 5 µM) or CBD (1 or 5 µM). MTT assay showed that both CBG and CBD at the tested doses were not cytotoxic, as confirmed by transcriptomic analysis that evidenced the downregulation of genes involved in cell death mechanisms such as *MAP3K5*, *PARP4, EIRF2AK3*, *ERN1, CASP6* and *CASP8* (Table 1). On the contrary, the data indicated that both compounds upregulated pro-survival genes, such as *AKT1*, *ATF4* and *BCL2L1*. In our analysis, treatment with CBG and CBD, especially at the concentration 5 µM, induced the upregulation of this gene. Moreover, CBG downregulated *CASP8* at low dose; while CBD downregulate *CASP6* and *CASP8* at 5 µM concentration. These results indicated that CBD at 5 µM is more efficacious in downregulating caspases. Regarding the pro-survival genes, *AKT1* encodes the serine-threonine protein kinase 1 (Akt1), belonging to Akt family, that promotes cellular survival, phosphorylating and inhibiting death-inducing proteins. Akt1 also regulates cell survival via the phosphorylation of MAP3K5, a kinase related to the apoptotic signal [19]. Therefore, Akt1-mediated phosphorylation of MAP3K5 reduces its activity and consequently prevents apoptosis. In compliance with this evidence, our results showed that CBG and CBD, at both doses, reduced the expression of *MAP3K5*. *BCL2L1* encodes a pro-survival protein belonging to the Bcl-2 family.

In this study, using NGS technology, we wanted only to analyze the transcriptomic profile of NSC-34 motor neuron-like cells treated with CBG or CBD at doses of 1 or 5 µM, in order to compare the effects of these two phytocompounds and evaluate how they modified the transcriptomic profile of the principal neurotransmission pathways. Our transcriptomic analysis demonstrated that treatment of NSC-34 motor neuron-like cells with CBG and CBD influenced several genes related to signaling pathways of the nervous system such as “Dopaminergic synapse”, “Glutamatergic Synapse” and “GABAergic synapse”. Specifically, CBG treatment modulates the same genes as CBD, inducing a similar transcriptomic profile.

Our results showed that in the “Dopaminergic synapse”, treatment with CBG and CBD downregulated the expression of *SLC18A*, *DRD2*, *GNAI*, *PRKCB*, *CAMK2*, *CREB1* and *CLOCK*. Conversely, both phytocompounds promoted the expression of seven genes (*DRD4*, *ADCY5*, *AKT1*, *ATF4*, *GNAI2*, *GNB4* and *PRKCA*). CBG downregulated the *SLC18A1* gene, more at the dose of 5 µM; while CBD strongly downregulated this gene at 1 µM dose. *SLC18A1* encodes for vesicular monoamine transporter 2, the principal of monoaminergic neurons. This transporter is present in the pre-synaptic vesicles of all monoaminergic neurons, and it is the common mechanism for the energy-driven uptake of dopamine [20]. It is worth noting that CBG downregulated the *DRD2* gene in a dose independent manner, while CBD downregulated this gene more at the highest dose. Conversely, both phytocompounds upregulated the *DRD4* gene, especially at the 5 µM dose. These genes encode Dopamine Receptor D2 and Dopamine Receptor D4, respectively. These receptors belong to the D2-like family and are associated with inhibiting G proteins (Gα_i/o_); therefore, they determine a reduction in cyclic adenosine monophosphate (cAMP) which causes the opening of potassium channels and closing of those of calcium. Furthermore, in our NSC-34 cells, CBG and CBD, in a dose-independent manner, downregulated the CREB1 gene, which encodes the cAMP-response element-binding protein (CREB). However, our transcriptional analysis showed that both phytocompounds upregulated *ATF4* gene, which also encodes for CREB2. CREB is located in the nucleus and is a transcription factor, which binds to the cAMP response element (CRE) of the promoters of its target genes. The protein is phosphorylated by various protein kinases such as mitogen-activated protein kinases (MAPK) and calmodulin-dependent protein kinase (CaMK) [21]. In our NSC-34 motor neuron-like cells, treatment with both phytocompounds reduced the expression of the *CAMK2B* gene, which encodes CaMK II Beta. CREB is involved in numerous intracellular processes, including proliferation, differentiation, survival, long-term synaptic enhancement, neurogenesis and neuronal plasticity [22]. Therefore, compounds such as CBG and CBD, by modulating the expression of this gene, could balance the phosphorylation levels of CREB.

Glutamate and GABA are two amino acid neurotransmitters that play an important role in controlling neuronal excitability [23]. To our knowledge, there are limited data about CBG influence on synaptic transmission. The CBG quinone derivative VCE-003.2 was reported to be able to reduce glial glutamate transporters [24]. On the contrary, CBD is known to be involved in the regulation of the excitatory transmission of glutamate and the inhibitory transmission of GABA [25]. In particular, CBD appears to be a GABA_A_ receptor modulator, being able to increase GABA-evoked currents [26].

Our results demonstrated that CBG and CBD upregulated the *GNAI2* gene. This gene encodes for the Guanine nucleotide-binding protein G (i) subunit alpha (α)-2. The guanine nucleotide-binding proteins are made of Gα, beta (β) and gamma (γ) subunits [27]. The α chain contains the guanine nucleotide-binding site and shows a GTPase activity that converts associated GTP to GDP, thus interrupting the signal. The Gβ and Gγ subunits form stable functional complexes that can regulate numerous effectors such as ion channels, enzymes and various kinases [28]. The β subunit is encoded by five genes (*GNB1-5*). In our transcriptomic analysis, CBD positively regulated *GNB4* in a dose-dependent manner. Conversely, the higher the dose of CBG, the less the gene is upregulated. Results from previous studies indicated that Gβ4 is widely expressed, can form dimers with nearly all Gγ subunits and modulated calcium, potassium channels and phospholipase isoforms [29]. β subunits of G proteins are associated with γ subunits, in order to form a Gβγ dimers, that affected glutamate transporter 1 (GLT-1) activity. In particular, GNB4 modulates the activity of glutamate transporter, suggesting a physical and functional interaction between GLT-1 and Gβγ dimers [30]. It has been shown that the subunits β2 are localized in the postsynaptic compartment, suggesting that Gβ-mediated signaling may be important for synapse formation and/or function [31].

The Gα_i/o_, including the subunit encoded by *GNAI2*, negatively couples with adenylyl cyclase (AC) with a consequent reduction in cAMP levels. A decrease in cAMP levels reduces protein kinase A (PKA) activity, reducing the release of neurotransmitters [32]. Our bioinformatic analysis, in association and according to the upregulation of *GNAI2*, reported that CBG and CBD downregulated, in a dose-dependent manner, the *ADCY9*. The *ADCY9* (adenylate cyclase 9) is the main gene involved in the signaling of the second messenger. Then, all together, these results may indicate that CBG treatment in NSC-34 motor neuron-like cells, and similarly CBD, may reduce the release of neurotransmitters through the G_i/o_-mediated inhibition of cAMP/PKA. Conversely, CBG increased the expression of *ADCY5*, more at the 1 µM dose compared to the 5 µM; while CBD upregulated *ADCY5* in a dose-dependent manner. *ADCY5* encodes for adenylate cyclase 5. The activity of *ADCY5* in motor neurons or in the peripheral nervous system remains to be clarified.

Our results showed that CBG and CBD also regulated genes involved in glutamate reuptake. After release from the presynaptic terminals, glutamate is quickly removed from the synaptic clefts by a family of five glutamate transporters, knows as excitatory amino acid transporters (EAAT1–5). EAATs are dual-function transport proteins: they are glutamate transporters and also act as anion channels [33]. CBG and CBD, at all doses, decreased the expression of the *SLC1A2* that encodes for EAAT2. The EAAT2 transporters are responsible for 90% of glutamate reuptake into the nervous system [34]. For these reasons, the modulation of EAATs in motor neurons by CBG and CBD is very interesting and allows the knowledge about phytocompound effects to be deepened.

Moreover, CBG downregulated, in a dose-dependent manner, the *SLC38A1* gene, downregulating it more at lower doses. Similarly, CBD modulates this gene. This gene encodes the glutamine transporter (GlnT) expressed in the membranes of glutamatergic neurons [35]. However, it has been shown that *SLC38A1* is also expressed in GABAergic neurons; therefore, it could be involved in the supply of the GABA neurotransmitter [36]. In this way, CBG and CBD might interact with the excitatory glutamatergic transmission.

In the postsynaptic compartment, CBG and CBD, at both doses, increased the expression of *DLGAP1* that encoded for Discs large associated proteins 1 (DLGAPs), also known as GKAP or SAPAP1. DLGAPs proteins that play an important role in the postsynaptic density are scaffold proteins and are involved in signaling glutamate receptors [37]. The DLGAP family may also have a role in modulating the turnover of ionotropic and metabotropic glutamate receptors in response to synaptic activity [38]. Therefore, DLGAPs are relevant for maintaining excitatory synapses. DLGAP proteins bind a second family of proteins, multiple ankyrin repeat domain proteins (SHANK) [39]. The SHANK family is characterized by a domain organization consisting of ankyrin repeats near the N terminal, followed by the SH3 domain, postsynaptic density protein-95, disk-large tumor suppressor protein, zonula occludens-1 (PDZ) domain, proline-rich region and a sterile alpha motif (SAM) domain at the C end. This organization allows them to mediate multiple interactions [40]. In our NSC-34 motor neuron-like cells, CBG downregulated the *SHANK1* gene more at the lower dose. Conversely, CBD downregulated this gene in a dose-dependent manner. SHANK proteins are expressed in the nervous system and are more concentrated in the excitatory synapses [40,41,42]. SHANK proteins are scaffold proteins that connect neurotransmitter receptors with other membrane proteins, with cytoskeleton or other protein to mediate the signal. These proteins mediate a platform needed for G-protein-mediated signaling. SHANK proteins are also involved in morphological changes, being involved in the maturation of dendritic spines and formation of the synapses [43]. These proteins play a key role in neurotransmission due to their ability to link metabotropic glutamate receptors (mGluRs) to ionotropic glutamate receptors, N-methyl-D-aspartate receptors (NMDARs) and α-amino-3-hydroxy-5-methyl- 4-isoxazole propionic acid receptors (AMPARs) [44,45]. Indeed, the PDZ domain of SHANK binds to the DLGAP family of proteins that in turn bind PSD-95, leading to the link of SHANK to the NMDA receptor [39]. Furthermore, SHANK binds also Homer which binds to group 1 and metabotropic glutamate receptors. Therefore, SHANK, due to its interactions with DLGAP and Homer, has the ability to link together the NMDA receptor complexes and the metabotropic glutamate receptor in the excitatory postsynaptic terminal [46]. DLGAP and SHANK are mutually dependent on each other, and these complexes play a key role in the correct positioning of many components in the excitatory postsynaptic terminal and the interactions between them [39]. For these reasons, the modulation of the DLGAP/SHANK pathway in NSC-34 motor neuron-like cells by CBG or CBD may alter the activity of the excitatory synapse and reduce the excitatory synaptic transmission. Indeed, it has been shown that the reduction in SHANK causes alterations of the DLGAP, NMDAR and AMPAR levels [47,48] with a consequent reduction in the number of synapses [46,49].

Interestingly, our results showed that CBG, and similarly CBD, negatively regulated the expression of the *PPP3R1* gene. However, conversely to CBD, CBG downregulated this gene more at the lower dose. The *PPP3R1* gene encodes for calcineurin, a serine/threonine phosphatase activated by calcium and calmodulin. This protein promotes the dephosphorylation of numerous biological targets, such as pro-apoptotic factors, transcription factors and ion channels [50]. Furthermore, it has been shown that it plays a key role in the apoptosis processes of neuronal cells, induced by neuroexcitotoxic stimuli due to the excessive release of glutamate [51]. In our analysis, CBG and CBD downregulated the *PRKCB* and *PLD1* genes encoding for protein kinase C (PKC) β and phospholipase D1 (PLD1), respectively. On the contrary, they slightly upregulated the *PRKCA* gene that encodes for PKCα. These proteins are involved in the downstream processes of the selective activation of mGluRs. Therefore, the strong downregulation of *PRKCB* and *PLD1* could reduce the postsynaptic glutamatergic activity. A novelty of this study is the finding that CBG can exert actions similar to CBD, reducing glutamate signaling in NSC-34 motor neuron-like cells.

Interestingly, our data also evidenced that CBG and similarly CBD influenced the gene expression involved in GABAergic transmission—specifically, CBG and CBD downregulation of the *SLC38A1* gene while they upregulated the *SLC32A1* gene. This gene encodes for solute carrier family 32 member 1, also known as vesicular GABA transporter, involved in the uptake of GABA into the synaptic vesicles. Our results demonstrate that CBG and CBD upregulating *SLC32A1* could potentiate inhibitory postsynaptic currents.

Our analysis demonstrated that NSC-34 motor neuron-like cells treated with CBG and with CBD showed the upregulation of *HAP1* gene, encoding Huntingtin-associated protein-1 (Hap1). This protein binds directly to the GABA A receptors, preventing their degradation on the plasma membrane with consequent improvement of inhibitory synaptic transmission [52]. Overexpression of Hap1 in cultured neurons has been shown to increase the number of GABA A receptors on the surface of the cell membrane, thereby increasing the average amplitude of the currents evoked from GABA [53]. In accordance with this evidence, our results may suggest that CBG and CBD, overexpressing the *HAP1*, may be able to reduce neuronal excitability in our motor neuron-like cells by enhancing postsynaptic GABAergic activity. However, these data should be confirmed in synaptosomal preparation and differentiated neuronal cells.

Furthermore, CBG and CBD can also regulate the phosphorylation of the GABA_A_ receptor through PKC. In line with our results, several studies have shown that different PKC isoforms can regulate the function of the GABA A receptor [54]. In this article, we have shown that CBD reduced the expression of genes encoding glutamine transporters. Conversely, CBD enhances GABA_A_ receptor activity. The main result of this work is that in our transcriptomic analysis CBG exhibited similar actions to CBD both for dopaminergic, GABAergic and glutamatergic signaling. CBG induced similar transcriptional changes in genes involved in dopamine, GABA and glutamate pathways compared to CBD.

## 4. Materials and Methods

### 4.1. Extraction and Isolation of CBG and CBD

*Cannabis sativa* was supplied by greenhouse cultivation at CREA-CIN (Rovigo, Italy) in compliance with their legal status (authorization SP/106 23/05/2013 of the Ministry of Health, Rome, Italy). CBG and CBD were isolated and purified (with greater than 99% purity) in agreement to a standardized protocol to avoid any trace of Δ^9^-THC [55,56].

### 4.2. Cell Culture and Cannabinoid Treatments

NSC-34 motor neuron-like cell line was purchased from Cellutions Biosystems Inc., Cedarlane (Burlington, ON, Canada). NSC-34 cells were cultured in DMEM-high glucose medium (Sigma-Aldrich, Saint Louis, MO, USA) supplemented with 10% FBS (Sigma-Aldrich, Saint Louis, MO, USA), at 37 °C in a moisturized atmosphere of 5% CO_2_ and 95% air. In order to evaluate the effects of CBG and CBD treatments, NSC-34 cells were treated for 24 h with various concentrations of CBG or CBD, namely 1 or 5 µM of CBG or CBD (DMSO < 0.1%). At the end of the treatment, cells were harvested for RNA extraction. All the experiments were performed in triplicate.

### 4.3. Thiazolyl Blue Tetrazolium Bromide (MTT) Assay

The effects of the treatment with CBG and CBD on cell viability were evaluated using the MTT assay. NSC-34 motor neuron-like cells were cultured in 96-well plates and incubated for 24 h with 1 or 5 µM CBG and 1 or 5 µM CBD. At the end of the treatment, cells were incubated with a fresh medium containing MTT (0.5 mg/mL; Sigma-Aldrich) at 37 °C for 4 h. Formazan crystals were dissolved in acidic isopropanol at 37 °C for 1 h, and the optical density was evaluated by spectrophotometric measurement of absorbance. The experiments were performed in triplicate.

### 4.4. Statistical Analysis of Cell Viability

The results are expressed by the mean ± SD. Statistical analysis of cell viability was performed using GraphPad Prism version 7.0 software (GraphPad Software, La Jolla, CA, USA). In order to test the normality of the data, the Shapiro–Wilk normality test was performed. All the groups appeared to be normally distributed against a *p*-value of 0.05. The multiple comparisons were carried out using one-way ANOVA test and Bonferroni posthoc test. A *p*-value less than or equal to 0.05 was considered statistically significant.

### 4.5. Total RNA Extraction and cDNA Library Preparation

RNA was obtained using the Maxwell^®^ RSC simplyRNA Cells Kit (Promega, Madison, WI, USA) following the manufacturer’s instructions. The library preparation was carried out according to the TruSeq RNA Exome protocol (Illumina, San Diego, CA, USA) following the instructions, as previously reported by Silvestro et al. [57].

### 4.6. Transcriptomic Analysis

The obtained libraries were then sequenced with an Illumina MiSeq Instrument. The quality of the raw data, stored in Fastq files, was confirmed by the fastQC tool, while Trimmomatic (version 0.38, Usadel Lab, Aachen, Germany) [58] was used to trimmer the bases with low quality and to remove the adapters. The cleaned reads were aligned to the reference genome of mouse organism GRCm38 and sorted using Spliced Transcripts Alignment to a Reference (STAR) RNA-seq aligner [59]. For each genome region, the reads identified were counter using python and, specifically, the package htseq-count [60]. The genes that differed in a statistical significant matter between CTR-NSC-34 and each treatment were obtained using the programming language R and the package DESeq2 of Bioconductor [61]. We rejected all the genes whose fold change was into the range of −0.7 and 0.7. Moreover, the Benjamini–Hochberg procedure was used to correct the *p*-value; only the genes with a *q*-value > 0.05 were analyzed.

## 5. Conclusions

Our transcriptomic analysis showed that CBG and CBD are able to influence the expression of the genes involved in glutamate, GABA and dopamine signaling. Interestingly, we found that CBG induced transcriptional changes in dopamine, GABA and glutamate pathways similar to CBD. Indeed, the genes upregulated by CBG were also upregulated by CBD, and at the same time, CBG and CBD downregulated the same genes. Transcriptomic analysis revealed that the two non-psychotropic cannabinoids reduced the expression of genes involved in glutamate release. Conversely, they enhanced the expression of genes involved in GABA release and improved the expression of the GABA A receptor. Moreover, CBG and CBD upregulated the expression of dopamine D4 receptor and of its downstream effectors. This transcriptomic analysis evidenced how CBG, compared to CBD, modified the electrophysiology of synaptic transmission of NSC-34 motor neuron-like cells. In addition, our study opens new perspective supporting future experiments in in vitro models of amyotrophic lateral sclerosis using differentiated NSC-34 motor neuron-like cells.

## Figures and Tables

**Table 1:** Differentially expressed genes up- and downregulated in apoptosis.

Gene	Name	Fold Change CBG 1 µM	Fold Change CBG 5 µM	Fold Change CBD 1 µM	Fold Change CBD 5 µM
*AKT1*	thymoma viral proto-oncogene 1	0.80	0.74	0.84	0.96
*ATF4*	activating transcription factor 4	0.71	0.74	0.80	0.76
*BCL2L1*	BCL2-like 1	0.97	1.20	0.96	1.29
*EIF2AK3*	eukaryotic translation initiation factor 2 alpha kinase 3	−1.84	−1.13	−1.82	−1.60
*ERN1*	endoplasmatic reticulum (ER) to nucleus signaling 1	−0.91	−0.91	−0.71	−0.84
*MAP3K5*	mitogen-activated protein kinase kinase kinase 5	−1.30	−0.97	−0.98	−0.82
*PARP4*	poly(ADP-ribose) polymerase family, member 4	−1.60	−1.59	−1.28	−1.50
*CASP6*	caspase 6	-	-	-	−3.58
*CASP8*	caspase 8	−1.39	-	-	−1.81

Each gene was associated with the fold change resulting in the analysis of CBG at dose of 1 and 5 µM against CTR-NSC-34 (“Fold Change CBG 1 µM” and “Fold Change CBG 5 µM”) and CBD at dose of 1 and 5 µM (“Fold Change CBD 1 µM” and “Fold Change CBD 5 µM”). The hyphen symbol indicates that the genes do not differ in a statistically significant way compared to CTR-NSC-34.

**Table 2:** Differentially expressed genes up- and downregulated related to Dopaminergic, GABAergic and Glutamatergic synapse pathways.

Gene	Name	Fold Change CBG 1 µM	Fold Change CBG 5 µM	Fold Change CBD 1 µM	Fold Change CBD 5 µM	Pathway
*ADCY5*	adenylate cyclase 5	5.86	4.74	5.25	6.03	Dopaminergic, GABAergic, Glutamatergic synapse
*ADCY9*	adenylate cyclase 9	−1.15	−1.28	−1.1	−1.31	GABAergic, Glutamatergic synapse
*AKT1*	thymoma viral proto-oncogene 1	0.80	0.74	0.84	0.96	Dopaminergic synapse
*ATF4*	activating transcription factor 4	0.71	0.74	0.80	0.76	Dopaminergic synapse
*CAMK2B*	calcium/calmodulin-dependent protein kinase II, beta	−1.33	−1.25	−1.02	−1.29	Dopaminergic synapse
*CLOCK*	circadian locomotor output cycles kaput	−1.59	−1.53	−1.41	−1.42	Dopaminergic synapse
*CREB1*	cAMP responsive element binding protein 1	−1.08	−0.98	−0.95	−1.23	Dopaminergic synapse
*DLGAP1*	DLG associated protein 1	1.61	1.56	1.32	1.26	Glutamatergic synapse
*DRD2*	dopamine receptor D2	−0.71	−0.74	−0.81	−1.08	Dopaminergic synapse
*DRD4*	dopamine receptor D4	1.61	2.03	1.36	1.84	Dopaminergic synapse
*GNAI2*	guanine nucleotide binding protein (G protein), alpha inhibiting 2	0.86	0.95	0.76	0.77	Dopaminergic, GABAergic, Glutamatergic synapse
*GNAL*	guanine nucleotide binding protein, alpha stimulating, olfactory type	−1.64	−5.79	−2.32	−1.74	Dopaminergic synapse
*GNB4*	guanine nucleotide binding protein (G protein), beta 4	1.46	1.14	0.74	1.16	Dopaminergic, GABAergic, Glutamatergic synapse
*HAP1*	huntingtin-associated protein 1	1.14	0.93	0.86	1.41	GABAergic synapse
*PLD1*	phospholipase D1	−1.12	−0.83	−2.39	−1.22	Glutamatergic synapse
*PPP3R1*	protein phosphatase 3, regulatory subunit B, alpha isoform (calcineurin B, type I)	−1.75	−1.45	−0.97	−1.7	Glutamatergic synapse
*PRKCA*	protein kinase C, alpha	0.97	1.09	0.86	0.95	Dopaminergic, GABAergic, Glutamatergic synapse
*PRKCB*	protein kinase C, beta	−2.71	−2.83	−4.81	−1.49	Dopaminergic, GABAergic, Glutamatergic synapse
*SHANK1*	SH3 and multiple ankyrin repeat domains 1	−1.22	−0.76	−0.86	−1.42	Glutamatergic synapse
*SLC1A2*	solute carrier family 1 (glial high affinity glutamate transporter), member 2	−0.84	−0.75	−0.72	−0.93	Glutamatergic synapse
*SLC18A1*	solute carrier family 18 (vesicular monoamine), member 1	−1.49	−3.61	−6.03	−1.58	Dopaminergic synapse
*SLC32A1*	solute carrier family 32 (GABA vesicular transporter), member 1	1.1	0.85	0.92	0.96	GABAergic synapse
*SLC38A1*	solute carrier family 38, member 1	−1.12	−0.91	−1.64	−1.33	GABAergic and Glutamatergic synapse

Each gene was associated with the fold change resulting in the analysis of CBG at dose of 1 and 5 µM against control (“Fold Change CBG 1 µM” and “Fold Change CBG 5 µM”) and CBD at dose of 1 and 5 µM (“Fold Change CBD 1 µM” and “Fold Change CBD 5 µM”). Furthermore, the column “Pathway” shows if the gene plays a role in the Dopaminergic, GABAergic or Glutamatergic synapse pathways.

## Supplementary Materials

The following are available online at https://www.mdpi.com/2075-1729/10/10/227/s1, Figure S1: Volcano Plot representation of the transcriptomic analysis of CBG at dose 1 µM (on the left) and 5 µM (on the right), Figure S2: Volcano Plot representation of the transcriptomic analysis of CBD at dose 1 µM (on the left) and 5 µM (on the right).

## Abbreviations

CBD	Cannabidiol
CBG	Cannabigerol
NGS	Next-generation sequencing
GABA	Gamma-aminobutyric acid
Δ^9^-THC	Delta-9-tetrahydrocannabinol
CBs	Cannabinoids recepors
GPCRs	G protein-coupled receptors
MTT	Thiazolyl Blue Tetrazolium Bromide
CTR-NSC-34	Control cells
DMSO	Dimethyl sulfoxide
*AKT1*	thymoma viral proto-oncogene 1
*ATF4*	activating transcription factor 4
*BCL2L1*	BCL2-like 1
*EIF2AK3*	eukaryotic translation initiation factor 2 alpha kinase 3
*ERN1*	endoplasmatic reticulum (ER) to nucleus signaling 1
*MAP3K5*	mitogen-activated protein kinase kinase kinase 5
*PARP4*	poly(ADP-ribose) polymerase family, member 4
*CASP6*	caspase 6
*CASP8*	caspase 8
*ADCY5*	adenylate cyclase 5
*DLGAP1*	DLG associated protein 1
*DRD4*	dopamine receptor D4
*GNAI2*	guanine nucleotide binding protein (G protein), alpha inhibiting 2
*GNB4*	guanine nucleotide binding protein (G protein), beta 4
*HAP1*	huntingtin-associated protein 1
*PRKCA*	protein kinase C, alpha
*SLC32A1*	solute carrier family 32 (GABA vesicular transporter), member 1
*ADCY9*	adenylate cyclase 9
*CAMK2B*	calcium/calmodulin-dependent protein kinase II, beta
*CLOCK*	circadian locomotor output cycles kaput
*CREB1*	cAMP responsive element binding protein 1
*DRD2*	dopamine receptor D2
*GNAL*	guanine nucleotide binding protein, alpha stimulating, olfactory type
*PLD1*	phospholipase D1
*PPP3R1*	protein phosphatase 3, regulatory subunit B, alpha isoform (calcineurin B, type I)
*PRKCB*	protein kinase C, beta
*SHANK1*	SH3 and multiple ankyrin repeat domains 1
*SLC1A2*	solute carrier family 1 (glial high affinity glutamate transporter), member 2
*SLC18A1*	solute carrier family 18 (vesicular monoamine), member 1
*SLC38A1*	solute carrier family 38, member 1
Akt1	serine-threonine protein kinase 1
α	Alpha
β	Beta
γ	Gamma
G α_i/o_	Inhibiting G proteins
AC	Adenylyl cyclase
cAMP	Cyclic adenosine monophosphate
CREB	cAMP-response element-binding protein
CRE	cAMP response element
MAPK	mitogen-activated protein kinases
CaMK	calmodulin-dependent protein kinase
PKA	Protein kinase A
EAATs	Excitatory amino acid transporters
GlnT	Glutamine transporter
DLGAPs	Discs large associated proteins 1
SHANK	Multiple ankyrin repeat domain proteins
PDZ	postsynaptic density protein-95, disk-large tumor suppressor protein, zonula occludens-1
SAM	sterile alpha motif
mGluRs	Metabotropic glutamate receptors
NMDARs	N-Methyl-D-aspartate receptors
AMPARs	α-amino-3-hydroxyl-5-methyl-4-isoxazole propionic acid receptors
PKC	Protein kinase C
PLD1	Phospholipase D1
HAT-1	Huntingtin-associated proteins-1
STAR	Spliced Transcripts Alignment to a Reference
