# Supplementary material for: The Transcriptomic Analysis of NSC-34 Motor Neuron-Like Cells Reveals That Cannabigerol Influences Synaptic Pathways: A Comparative Study with Cannabidiol
Source: Life (Basel). 2020 Oct 1;10(10):227. doi: 10.3390/life10100227 (PMC7600552; doi:10.3390/life10100227)
Supplement: Supplementary file 1 [file life-10-00227-s001.pdf]

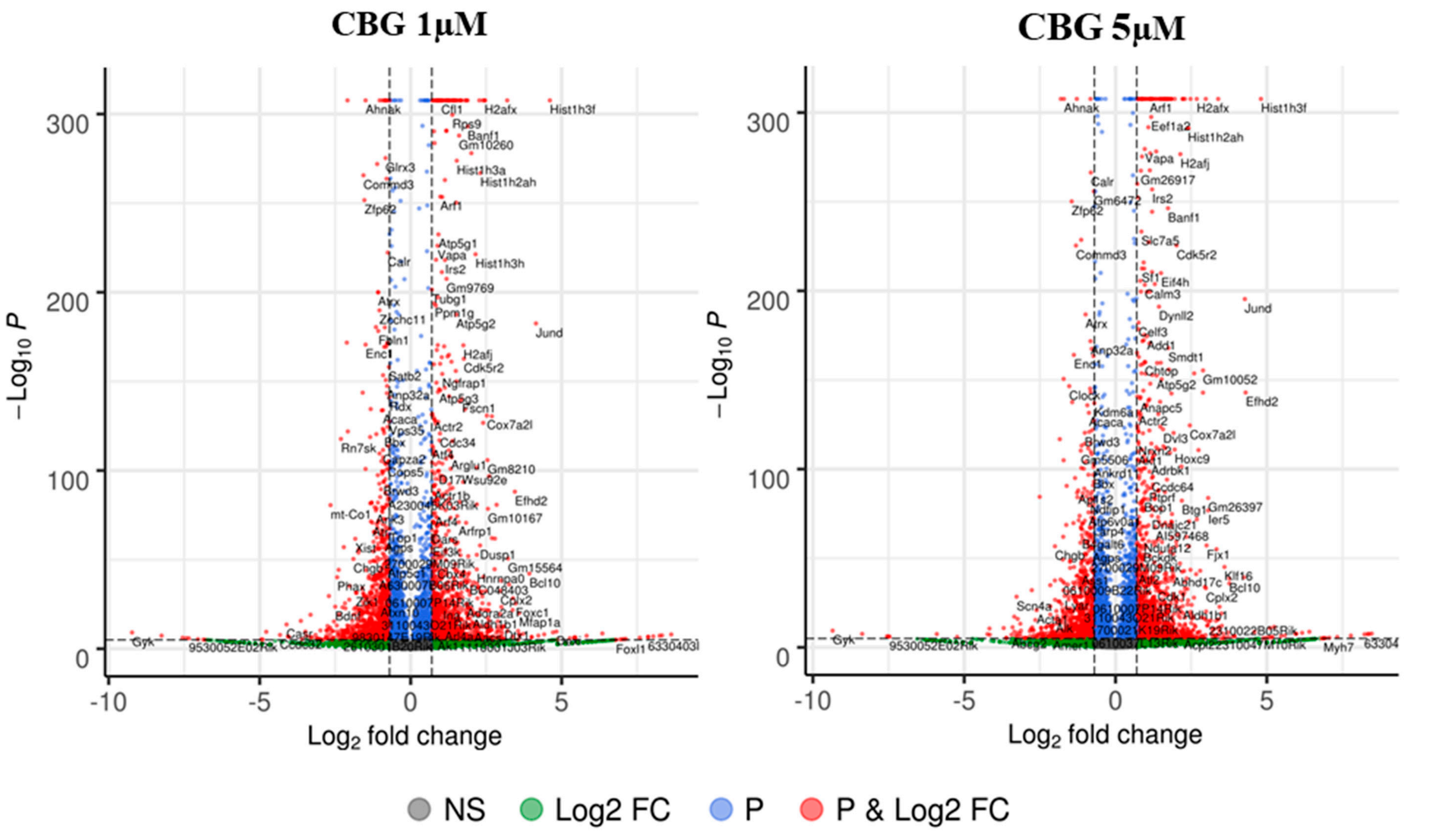

Figure S1. Volcano Plot representation of the transcriptomic analysis of CBG at dose 1 μM (on the left) and 5 μM (on the right). All the genes found in the analysis were reported here. The grey points (NS) stands for genes that have a p-value higher than 0.05 and a fold change lower than 0.7. The green points (Log2 FC) are the genes with fold change higher than 0.7 but with a p-value higher than 0.05. Conversely, the blue points represent the genes which fold change is lower than 0.7 but their p-value is lower than 0.05. The genes depicted by red points have a p-value lower than 0.05 and a fold change higher than 0.7.

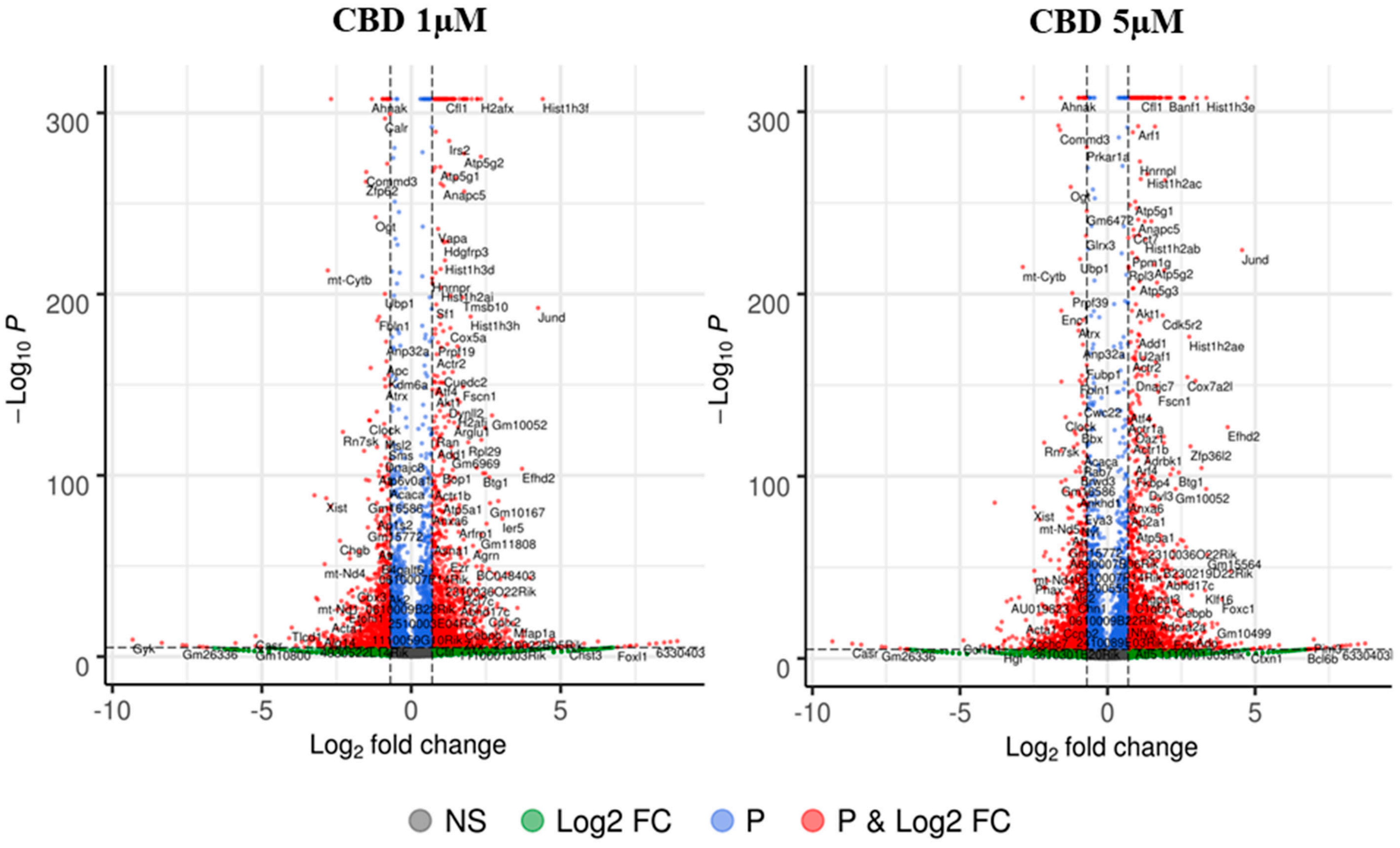

Figure S2. Volcano Plot representation of the transcriptomic analysis of CBD at dose 1 µM (on the left) and 5 µM (on the right). All the genes found in the analysis were reported here. The grey points (NS) stands for genes that have a p-value higher than 0.05 and a fold change lower than 0.7. The green points (Log2 FC) are the genes with

fold change higher than 0.7 but with a p-value higher than 0.05. Conversely, the blue points represent the genes which fold change is lower than 0.7 but their p-value is lower than 0.05. The genes depicted by red points have a p-value lower than 0.05 and a fold change higher than 0.7.
